# Recreation of in-host acquired single nucleotide polymorphisms by CRISPR-Cas9 reveals an uncharacterised gene playing a role in *Aspergillus fumigatus* azole resistance via a non-*cyp51A* mediated resistance mechanism

**DOI:** 10.1016/j.fgb.2019.05.005

**Published:** 2019-09

**Authors:** Eloise Ballard, Jakob Weber, Willem J.G. Melchers, Seshu Tammireddy, Phillip D. Whitfield, Axel A. Brakhage, Alistair J.P. Brown, Paul E. Verweij, Adilia Warris

**Affiliations:** a MRC Centre for Medical Mycology at the University of Aberdeen, Aberdeen Fungal Group, Institute of Medical Sciences, University of Aberdeen, UK; b Department of Molecular and Applied Microbiology, Leibniz Institute for Natural Product Research and Infection Biology – Hans Knöll Institute, Jena, Germany; c Centre for Expertise in Mycology and Department of Medical Microbiology, Radboud University Medical Centre, Nijmegen, the Netherlands; d Lipidomics Research Facility, Division of Biomedical Sciences, University of the Highlands and Islands, UK

**Keywords:** *Aspergillus fumigatus*, Azole resistance, CRISPR-Cas9, In-host adaptation, Ergosterol

## Abstract

The human host comprises a range of specific niche environments. In order to successfully persist, pathogens such as *Aspergillus fumigatus* must adapt to these environments. One key example of in-host adaptation is the development of resistance to azole antifungals. Azole resistance in *A. fumigatus* is increasingly reported worldwide and the most commonly reported mechanisms are *cyp51A* mediated. Using a unique series of *A. fumigatus* isolates, obtained from a patient suffering from persistent and recurrent invasive aspergillosis over 2 years, this study aimed to gain insight into the genetic basis of in-host adaptation. Single nucleotide polymorphisms (SNPs) unique to a single isolate in this series, which had developed multi-azole resistance in-host, were identified. Two nonsense SNPs were recreated using CRISPR-Cas9; these were 213^*^ in *svf1* and 167^*^ in uncharacterised gene AFUA_7G01960. Phenotypic analyses including antifungal susceptibility testing, mycelial growth rate assessment, lipidomics analysis and statin susceptibility testing were performed to associate genotypes to phenotypes. This revealed a role for *svf1* in *A. fumigatus* oxidative stress sensitivity. In contrast, recapitulation of 167^*^ in AFUA_7G01960 resulted in increased itraconazole resistance. Comprehensive lipidomics analysis revealed decreased ergosterol levels in strains containing this SNP, providing insight to the observed itraconazole resistance. Decreases in ergosterol levels were reflected in increased resistance to lovastatin and nystatin. Importantly, this study has identified a SNP in an uncharacterised gene playing a role in azole resistance via a non-*cyp51A* mediated resistance mechanism. This mechanism is of clinical importance, as this SNP was identified in a clinical isolate, which acquired azole resistance in-host.

## Highlights

• New in-host acquired non-*cyp51*-mediated azole resistance mechanism in *A. fumigatus.*
• 167^*^ mutation in AFUA_7G01960 leads to azole resistance and thermotolerance.
• Azole resistance associated with decreased ergosterol content in fungal membrane.

## 1 Introduction

In order to survive in-host, the human fungal pathogen *Aspergillus fumigatus* must adapt to specific niche environments (Verweij et al., 2016). Adaptation can occur as a result of spontaneous mutation and subsequent selection. One clinically important example of in-host adaptation is the development of resistance to azole antifungals. Azole resistance in *A. fumigatus* is being increasingly reported globally (Denning and Perlin, 2011). A range of mutations in the *cyp51A* gene, which encodes the ergosterol biosynthetic enzyme lanosterol 14-alpha-demethylase, have been shown to cause azole resistance by altering the structure of ligand access channels. Amino acid changes in these channels are thought to disturb docking of azole molecules by altering interactions (Snelders et al., 2010). These include mutations such as P216, G138, M220 and G54 (Albarrag et al., 2011, Garcia-Effron et al., 2005, Mann et al., 2003, Mellado et al., 2004, Snelders et al., 2010). In addition, tandem repeats in the promoter region of *cyp51A,* in combination with L98H within the *cyp51A* coding region, have been shown to confer itraconazole resistance (Gsaller et al., 2016, Mellado et al., 2007, Snelders et al., 2008, Zhang et al., 2017). Few non-*cyp51A* mediated resistance mechanisms have been described in *A. fumigatus* thus far. One example is P88L in the HapE subunit of the CCAAT-binding complex, which is a multimeric transcription factor acting as a negative regulator of ergosterol biosynthesis (Camps et al., 2012, Gsaller et al., 2016). Other proteins reported to be associated with azole resistance include RamA (farnesyltransferase β-subunit), mitochondrial complex I and efflux transporters AtrF and Cdr1B (Bromley et al., 2016, Fraczek et al., 2013, Norton et al., 2017, Slaven et al., 2002).

We have previously shown that *A. fumigatus* undergoes significant in-host adaptation using a large series of isogenic strains, isolated from a single patient over a two year period (Ballard et al., 2018, Verweij et al., 2016). Phenotypic analyses revealed that these isogenic strains developed in-host azole antifungal resistance, changes in colony morphology, defects in conidiation, and alterations in virulence (Ballard et al., 2018). Whole genome comparisons identified 248 non-synonymous single nucleotide polymorphisms (SNPs) that were absent in the early isolates but present in strains subsequently isolated from the patient. We hypothesise that these SNPs could potentially play a role in the observed in-host adaptation and possibly enable persistent infection (Ballard et al., 2018). Here we extend our previous study by investigating the phenotypic impact of selected SNPs using a newly developed CRISPR-Cas9 method to recapitulate specific SNPs observed in clinical *A. fumigatus* strains.

In this study, we focused on one particular strain of interest from our series of 13 isogenic strains. This strain, V157-62, was multi-azole resistant (MIC itraconazole > 16 mg/L, voriconazole 8 mg/L and posaconazole > 16 mg/L), harboured M220R in *cyp51A* which partially explained its resistance profile (Chen et al., 2005, Garcia-Effron et al., 2005, Mellado et al., 2004) and also showed a severe growth defect (Ballard et al., 2018). Unique to this strain were 14 non-synonymous SNPs in 8 genes: 12 of these were missense variants, of which the impact on the encoded protein is unclear. However, 2 SNPs generated premature stop codons, which could result in loss, or at least perturbation, of protein function. One SNP was 213^*^ identified in *svf1*, which is known to play a role in oxidative stress responses in *Saccharomyces cerevisiae* (Brace et al., 2005). The second SNP was 167^*^ identified in an uncharacterised gene (AFUA_7G01960). These 2 nonsense mutations were selected for further investigation using CRISPR-Cas9 and were recreated in the first isolate of the series (V130-15). Our phenotypic analyses showed that one SNP influenced oxidative stress sensitivity. Interestingly, we also showed the role of an in-host acquired SNP in an uncharacterised gene resulting in increased azole resistance. Using comprehensive lipidomics analysis this SNP was associated with decreased ergosterol levels. These data provide evidence for a novel in-host acquired non-*cyp51*-mediated azole resistance mechanism in *A. fumigatus.*

## 2 Materials and methods

### 2.1 Origin of fungal isolates

We focused on *A. fumigatus* strain V157-62, which is one of the isolates from a series of 13 strains isolated from a patient diagnosed with X-linked chronic granulomatous disease who suffered from recurrent/persistent aspergillosis over a period of 2 years (Ballard et al., 2018, Verweij et al., 2016). In addition, two isolates from the same patient V130-15 (azole-susceptible phenotype) and V157-39 (azole-resistant phenotype with G54R mutation in *cyp51A*) were used for molecular experimentation. All strains have previously been phenotypically and genotypically characterised by us (Ballard et al., 2018).

Firstly, as a control of the CRISPR-Cas9 method, we reversed the G54R mutation in *cyp51A* to WT in V157-39. Then we focused on SNPs identified in V157-62. Whole genome comparisons previously performed, identified several SNPs unique to V157-62 (Ballard et al., 2018). Two of these SNPs were selected for further investigation as they may result in protein truncations. These SNPs were 213^*^ in *svf1* (survival factor 1; AFUA_5G11820) and 167^*^ in the uncharacterised gene, AUFA_7G01960. These SNPs were recreated in the azole susceptible strain V130-15 (the first strain isolated in the series). This approach was selected as it enabled the phenotypes associated with each SNP to be investigated individually in a susceptible clinical isolate, with an isogenic genetic background to the isolate in which the SNPs were identified, but with a wild-type growth phenotype.

Strains used in this study are shown in Table 1. Also summarised in Table 1 are previously published isolation dates, genotypes and the number of non-synonymous SNPs different between each isolate (Ballard et al., 2018).

**Table 1:** Description of *A. fumigatus* isolates in the study including minimum inhibitory concentrations.

Isolation date	Strain	Genotype	Number of non-synonymous single nucleotide polymorphisms different	Minimum inhibitory concentration (MIC; mg/L)			
				Itraconazole	Voriconazole	Posaconazole	Amphotericin B
22/11/11	V130-15	WT *cyp51A*		1	1	0.25	0.5
	V130-15*svf1*^213*^	213^*^ in *svf1*		1	1	0.25	0.5
	V130-15*unc*^167*^	167^*^ in AFUA_7G01960		**4**	2	0.25	0.5
9/12/13	V157-39*cyp51A*^G54R^	G54R in *cyp51A*	21 different from V130-15 35 different from V157-62	**>16**	1	**>16**	0.5
	V157-39*cyp51A*^WT^	WT *cyp51A*		0.5	1	**>16**	0.5
9/12/13	V157-62	M220R in *cyp51A;* 213^*^ in *svf1*; 167^*^ in AFUA_7G01960	29 different from V130-15 35 different from V157-39	**>16**	**8**	**>16**	0.25

Bold indicates a MIC exceeding the EUCAST clinical resistance breakpoint; which are defined as itraconazole > 2 mg/L, voriconazole > 2 mg/L, posaconazole > 0.25 mg/L and amphotericin B > 2 mg/L.

### 2.2 Molecular techniques

#### 2.2.1 General molecular techniques

Plasmids were designed using Benchling (www.benchling.com). All PCR reactions were performed using Phusion Flash High-Fidelity PCR Master Mix (Thermo Fisher Scientific, UK) and purified using the QIAquick® PCR purification kit (Qiagen, UK). Sanger sequencing was performed using the Lightrun service (GATC Biotech, Germany) and analysed using GATCViewer software. DNA was quantified using a Nanodrop 1000 (Thermo Fisher Scientific, USA).

#### 2.2.2 Genomic DNA extraction

Fungal strains were grown overnight in liquid glucose minimal media at 37 °C and 200 rpm. Genomic DNA was extracted from the mycelium using the DNeasy Plant Mini Kit (Qiagen, Germany). Mycelia were disrupted in the presence of AP1 buffer, RNAse and glass beads using a FastPrep-24 (MP Biomedicals, UK) at max speed for 1 min. The kit DNA extraction procedure was subsequently followed.

#### 2.2.3 CRISPR-Cas9 preparation

**Fig. A.1:**
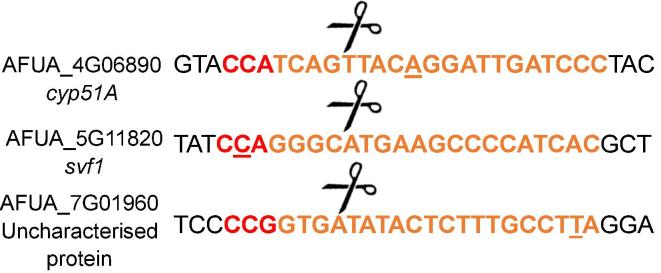
Schematic showing the protospacer adjacent motif (PAM), protospacer and Cas9 cut site for each target. Colour scheme is as follows: red text represents 3 bp PAM site, orange text represents 20 bp protospacer, scissors represent Cas9 cut site and underlining indicates the location of the target nucleotide for modification.

We used a method adapted from previously published methods (Nødvig et al., 2015, Weber et al., 2016). Two plasmids and donor DNA containing the desired SNP sequence were used. Plasmid 1 encoded AMA1, Cas9, hygromycin resistance and ampicillin resistance. The second plasmid, which was target specific, encoded the guide RNA, hammerhead ribozyme, protospacer, ampicillin resistance and split-pyrithiamine resistance. Protospacer adjacent motif (PAM) and protospacer sequences near to the target site were identified manually using publicly available sequences obtained from AspGD (www.aspgd.org). Selected PAM and protospacer sequences are shown in Fig. A.1. Plasmid 2 was created for each individual target according to the previously published method (Nødvig et al., 2015). Using a plasmid template and overlapping PCR primer pairs, fragments of the target specific plasmid could be obtained. DpnI digestion was used to remove residual plasmid template. Gibson assembly was used to ligate plasmid fragments and was performed using the NEBuilder® HiFi DNA Assembly Master Mix (New England Biolabs, UK). Plasmids were propagated using subcloning efficiency DH5α *Escherichia coli* (Invitrogen, UK) grown on LB agar with 100 µg/ml ampicillin (Sigma Aldrich, UK). Donor DNA was produced by PCR using genomic DNA from strains containing the target SNP. Primers used are shown in Table A.1.

#### 2.2.4 CRISPR-Cas9 transformation

Three clinical isolates are utilised in this study. Strain V130-15 was the initial clinical isolate and V157-39 was a subsequent isolate that evolved azole resistance in-host. Isolate V157-62 was a later isolate which has acquired azole resistance and a growth defect in-host; this isolate was used to identify SNPs (see Table 1). The SNPs identified in V157-62 were recreated in the initial isolate V130-15.

*A. fumigatus* cultures grown overnight in liquid glucose minimal media at 37 °C and 200 rpm were harvested using a 40 µm strainer. Protoplasts were made using a modification of a published protocol (Ballance et al., 1983, Ballance and Turner, 1985). The fungal biomass was transferred into an Erlenmeyer flask containing protoplasting solution consisting of Vinotaste Pro (Novozymes, Denmark) dissolved in Trafo I (0.6 M KCl, 10 mM Na_2_PO_3_, pH 5.8). The flask was incubated at 30 °C and 80 rpm for 3 h; every 30 min the solution was resuspended using a serological pipette. Protoplast formation was monitored using a light microscope, once formed, protoplasts were filtered using 40 µm strainer washed with Trafo I. Protoplasts were centrifuged at 4 °C and 4000 rpm for 5 min. The pellet was resolved in Trafo II (0.6 M KCl, 100 mM Tris, pH 7) and washing was repeated. The pellet was subsequently resolved in Trafo III (0.6 M KCl, 10 mM Tris, pH 7.5). Protoplasts were transformed on ice using PEG solution (25% PEG 8000 in Trafo III), 3 µg of each plasmid and 1 µg donor DNA. Transformants were mixed into glucose minimal agar containing 1 M sorbitol, 1 µg/ml pyrithiamine hydrobromide (Sigma Aldrich, UK) and 100 µg/ml hygromycin B (Roche, UK) and incubated at 37 °C. Colonies were streak purified with and then without selection markers. It is important to note that without selection, plasmid 1 (which encodes AMA1 and Cas9) is readily lost from the fungus, preventing further gene editing from occurring in the strain after streak purification (Aleksenko and Clutterbuck, 1997, Nødvig et al., 2015). Single nucleotide changes were verified using PCR and Sanger sequencing.

### 2.3 Phenotypic analysis

#### 2.3.1 Conidial suspension preparation

*A. fumigatus* conidia were spread onto Sabouraud dextrose agar in T75 culture flasks (Greiner Bio-One, Germany) and incubated at 37 °C for 7 d. Conidia were harvested via immersion in phosphate buffered saline (PBS) (Thermo Fisher Scientific, UK) containing 0.05% Tween-80 (Thermo Fisher Scientific, UK). Conidial suspensions were filtered using a 40 µm strainer, washed twice using PBS and counted using a Neubauer improved haemocytometer.

#### 2.3.2 Susceptibility testing

*In vitro* susceptibility testing of the isolates against antifungals and statins was performed according to the EUCAST broth microdilution reference method (EUCAST, 2015). Nystatin (Sigma Aldrich, UK) and lovastatin (Sigma Aldrich, UK) were solubilized in dimethyl sulfoxide (DMSO), diluted in double strength RPMI and tested at final concentrations of 16 µg/ml and 256 µg/ml respectively. After incubation with statins for 48 h, an XTT (2,3-bis(2-methoxy-4-nitro-5-sulfophenyl)-2H-tetrazolium-5-carboxanilide) assay was performed to quantify fungal viability (Meletiadis et al., 2001). Susceptibility testing was performed in duplicate with 3 technical repeats per experiment.

#### 2.3.3 Mycelial growth rate analysis

Sabouraud dextrose agar plates were spot-inoculated with 5x10^2^ conidia. Selected plates were supplemented with 2.5 mM H_2_O_2_. Plates were incubated at 37 °C (unless otherwise indicated) for 96 h. Colony diameters were measured every 24 h. Each condition was performed in duplicate with 3 technical repeats per experiment.

#### 2.3.4 In silico analysis of uncharacterised protein

Exon only nucleotide and amino acid sequences were obtained from AspGD (www.aspgd.org/) and UniProt (www.uniprot.org). Sequences were used in NCBI conserved domain search and BLAST analyses (Altschul et al., 1990, Marchler-Bauer et al., 2015). Phyre^2^ was used for protein structure modelling and analysis (Kelly et al., 2015).

#### 2.3.5 Sterol and phosphatidylinositol quantification

Fungal strains (V130-15, V130-15*unc*^167*^, and V157-62) were grown overnight in liquid glucose minimal media in Erlenmeyer flasks at 37 °C and 200 rpm. The fungal mass was collected via vacuum filtration and dried at 60 °C for 2 h. Dried mycelia were grinded in a pestle and mortar using liquid nitrogen, this process was repeated until a fine powder was reached. The powder was stored in absolute ethanol until extraction.

For ergosterol quantification, fungal samples were hydrolysed with 0.1 M ethanolic KOH (Thermo Fisher Scientific, UK) containing 5α-cholestane (Sigma Aldrich, UK) as an internal standard. Ergosterol was extracted in hexane (Thermo Fisher Scientific, UK), dried under nitrogen and derivatised with MSTFA + 1% TCMS (Thermo Fisher Scientific, UK). Total (free and esterified) ergosterol concentrations were measured by gas chromatography-mass spectrometry (GC–MS). GC–MS analysis was performed using a Thermo Trace Ultra gas chromatograph fitted with a DB-5 ms column (30 m × 0.25 mm i.d. × 0.25 µm film; Agilent J&W, USA) and coupled to a Thermo ISQ mass spectrometer. Samples were injected (1 µl) in splitless mode using helium as a carrier gas (1 ml/min). After a delay of 2 min at 150 °C, the temperature was ramped to 300 °C at 15 °C/min and then held for 8 min. The mass spectrometer was operated in selected ion monitoring (SIM) mode using mass to charge ratio (*m*/*z*) 372 for 5α-cholestane and *m*/*z* 468 for ergosterol.

For phosphatidylinositol quantification, extractions were performed according to a published method (Folch et al., 1957). Phosphatidylinositol 32:0 (Avanti Polar Lipids, USA) was included in the experimental system as an internal standard. The samples were analysed by liquid chromatography-mass spectrometry (LC-MS) using a Thermo Exactive Orbitrap mass spectrometer interfaced to a Thermo Accela 1250 UHPLC system. Samples were injected (2 µl) onto a Hypersil Gold C18 column (2.1 mm × 100 mm, 1.9 μm; Thermo Fisher Scientific, UK) maintained at 50 °C. Mobile phase A consisted of water containing 10 mM ammonium formate and 0.1% (v/v) formic acid. Mobile phase B consisted of 90:10 isopropanol/acetonitrile containing 10 mM ammonium formate and 0.1% (v/v) formic acid. The initial conditions for analysis were 65%A/35%B. The percentage of mobile phase B was increased from 35% to 65% over 4 min, followed by 65%-100% over 15 min held for 2 min before re-equilibration to the starting conditions over 6 min. The flow rate was 400 µl/min. The mass spectrometer was operated in negative ion mode over the mass to charge ratio (*m*/*z*) range of 250–2000 at a resolution of 100,000. Ion signals corresponding to the accurate *m*/*z* values for [M−H]^−^ ions of phosphatidylinositol 34:2 (*m*/*z* 833.5186) and phosphatidylinositol 32:0 (*m*/*z* 809.5201) were extracted from raw LC–MS data sets with the mass error set to 5 ppm.

Ergosterol and phosphatidylinositol were measured from the same samples and measurements were normalised to mg dry weight. Two independent experiments were performed, comprising a total of 6 biological repeats per strain and 3 technical repeats per sample.

### 2.4 Ethics statement

All *A. fumigatus* strains used in this study were obtained from the culture collection at the Department of Medical Microbiology, Radboud University Medical Centre. All strains were anonymised.

### 2.5 Statistical analysis

All statistical analysis was performed using GraphPad Prism (version 5). Statistical significance was assessed using a two-tailed T test; a p value < 0.05 was considered significant.

## 3 Results

### 3.1 Reversal of G54R in cyp51A using CRISPR-Cas9

**Fig. 1:**
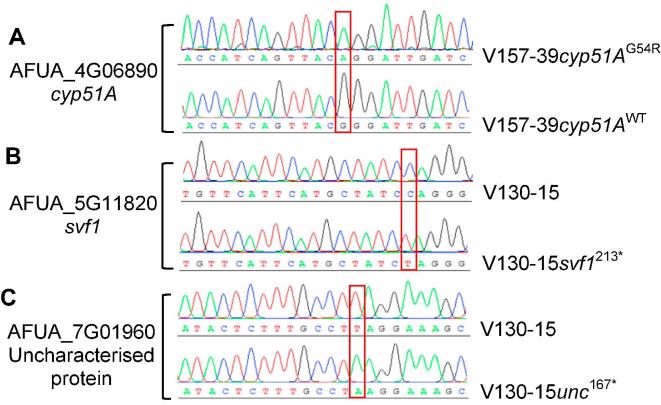
Verification of SNP generation via Sanger sequencing**.** (A) Verification of A → G in *cyp51A* (AFUA_4G06890). (B) Verification of C → T in *svf1* (AFUA_5G11820) (C). Verification of T → A in uncharacterised protein (AFUA_7G01960).

First, we wanted to establish that CRISPR-Cas9 can be used to recapitulate SNPs that affect antifungal drug resistance in clinical isolates of *A. fumigatus.* To do this, we reversed the G54R mutation in *cyp51A* to wild type (AGG → GGG) in V157-39. This mutation was then verified by Sanger sequencing (Fig. 1A). The azole resistance phenotype of V157-39 included itraconazole > 16 mg/L, voriconazole 1 mg/L and posaconazole > 16 mg/L. Reversal of G54R to WT in *cyp51A* reduced itraconazole resistance (MIC > 16–0.5 mg/L) but had no effect on posaconazole resistance (MIC > 16 mg/L). Reversal of G54R did not result in any alterations in mycelial growth rate or colony morphology. MICs are summarised in Table 1.

### 3.2 Recreation of 213^*^ in svf1

To examine the contribution of the 213^*^ SNP in *svf1* to the phenotype of isolate V157-62, we recreated 213^*^ (C → T) in *svf1* (AFUA_5G11820), which was identified in V157-62, in V130-15, the first isolate of the series (verification shown in Fig. 1B). This mutation had no impact on azole resistance but increased the susceptibility of the resultant *A. fumigatus* strain (V130-15*svf1*^213*^) to H_2_O_2_. MICs are shown in Table 1.

**Fig. 2:**
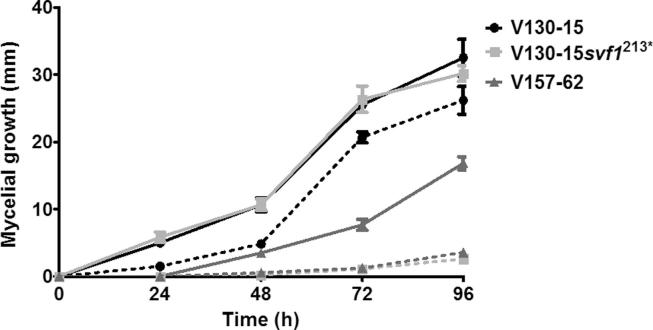
Mycelial growth of *A. fumigatus* strains V130-15, V130-15s*vf1*^213*^ and V157-62 with or without H_2_O_2_. Solid lines indicate growth in the absence of H_2_O_2_; dotted lines indicate growth in the presence of 2.5 mM H_2_O_2._ Sabouraud dextrose agar plates were spot inoculated with 5x10^2^*Aspergillus* conidia in 5 µl PBS and cultured at 37 °C. Colony diameter was measured every 24 h. Data represents two independent experiments with 3 repeats per experiment. Error bars represent ± SD.

As shown in Fig. 2, the growth of all three strains was reduced in the presence of 2.5 mM H_2_O_2_ (e.g. p < 0.0001 for V130-15). However, the growth of those strains that possess 213^*^ in *svf1* (V130-15*svf1*^213*^ and V157-62) was affected to a much greater extent by the presence of 2.5 mM H_2_O_2_ (p < 0.001). In comparison to when grown under control conditions, strains V130-15*svf1*^213*^ and V157-62 showed reductions in 96 h colony diameters when grown with 2.5 mM H_2_O_2_ of 27.5 mm and 13.2 mm respectively. Interestingly, the 96 h colony diameter of V130-15 with 2.5 mM H_2_O_2_ was on average only 5 mm smaller than when grown under control conditions. Therefore, the 213^*^ SNP in *svf1* confers sensitivity H_2_O_2_.

### 3.3 Recreation of 167^*^ in an uncharacterised protein

As shown in Fig. 1C, the SNP 167^*^ (T → A) in the uncharacterised gene AFUA_7G01960, which was identified in V157-62, was recreated in isolate V130-15. This resulted in increased resistance to itraconazole (MIC 1 to 4 mg/L), but minimal difference to voriconazole resistance (MIC 1 to 2 mg/L). This had no effect on posaconazole or amphotericin B susceptibility (MICs 0.25 mg/L and 0.5 mg/L, respectively). MICs are displayed in Table 1.

**Fig. 3:**
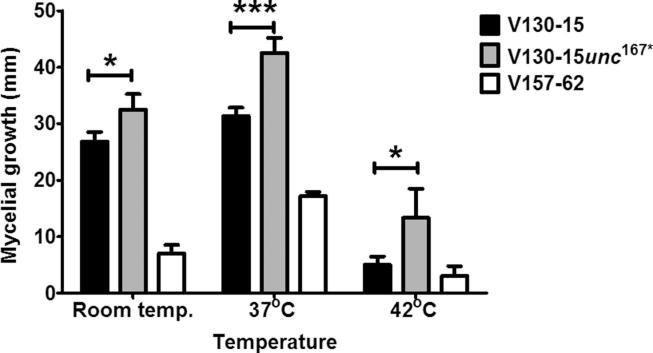
Mycelial growth of *A. fumigatus* strains V130-15, V130-15*unc*^167*^ and V157-62 at different temperatures. Sabouraud dextrose agar plates were spot inoculated with 5x10^2^*Aspergillus* conidia in 5 µl PBS and cultured at different temperatures. Colony diameter was measured after 96 h. Data were obtained in two experiments each comprising triplicate repeats. Mean values ± SD are shown (^*^p < 0.01; ^***^p < 0.0001; two-tailed Students T-test).

**Fig. 4:**
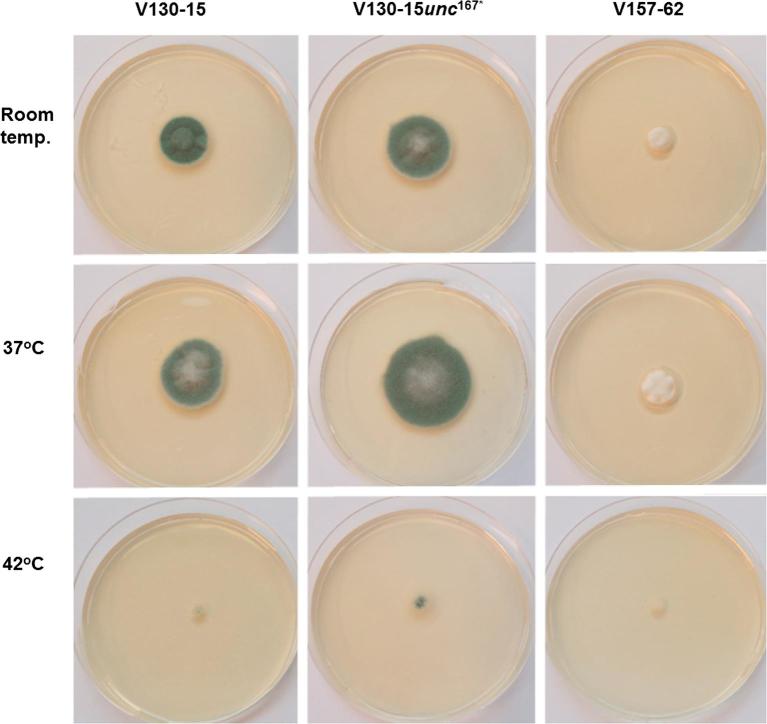
Morphology and growth differences of *A. fumigatus* strains V130-15, V130-15*unc*^167*^ and V157-62 at different temperatures. Sabouraud dextrose agar plates were spot inoculated with 5 × 10^2^*Aspergillus* conidia in 5 µl PBS and cultured. Images were taken after 96 h.

As shown in Fig. 3, Fig. 4, V130-15*unc*^167*^ showed temperature dependent differences in mycelial growth after 96 h. V130-15*unc*^167*^ grew faster than its parental strain V130-15 at room temperature (32.5 and 26.8 mm, respectively (p < 0.01)). This difference became even more pronounced at 37 °C, with a mean colony diameter of the V130-15*unc*^167*^ strain of 42.5 mm and 31.3 mm for the parent strain (p < 0.0001). Growth at 42 °C showed a comparable picture, with the transformed strain displaying a mean colony diameter 8.33 mm greater than the parent V130-15 (p < 0.01). Under all conditions tested, V157-62 had smaller mean colony diameter in comparison to both the parent and transformant.

In order to gain insight into the possible mechanisms facilitating these phenotypes we performed *in silico* analyses into this uncharacterised protein encoded by AFUA_7G01960. NCBI conserved domain searching identified 2 conserved domains; a fungal transcription factor middle homology region (E value = 5 × 10^−324^) and a GAL-4-like C6 zinc binuclear cluster DNA binding domain (E value = 5.72 × 10^−13^). BLAST searching identified 138 orthologues for the gene among 22 different *Aspergillus* spp; this gene shared 94.8% identity with *Aspergillus udagawae* sterol uptake control protein 2 (E value = 5 × 10^−324^). Additionally, structure modelling (using Phyre^2^) suggested that the AFUA_7G01960 protein shares 36% identity with the crystal structure of sterol uptake control protein 2 (*upc2*) in *S. cerevisiae* (p = 2 × 10^−10^) (Ha et al., 2013, Yang et al., 2015)*.*

**Fig. 5:**
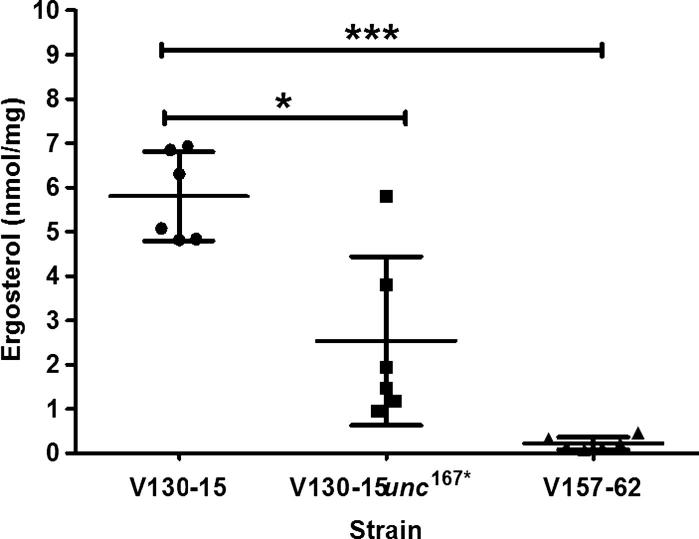
Quantification of total ergosterol content of *A. fumigatus* strain V130-15, V130-15*unc*^167*^ and V157-62. Strains were cultured overnight in liquid glucose minimal media at 37 °C shaking at 200 rpm. Fungal mass was dried and ground in liquid nitrogen. Samples were subjected to alkaline hydrolysis and ergosterol was solvent extracted and derivatised. Ergosterol was then quantified using gas chromatography-mass spectrometry (GC–MS). Data represents two independent extraction experiments, comprising 6 biological repeats per strain and 3 technical repeats per sample. Error bars represent ± SD (^*^p < 0.01; ^***^p < 0.0001; two-tailed Students T-test).

Overall, our *in silico* analyses suggested the uncharacterised gene, AFUA_7G01960, shares similarities with genes involved in sterol regulation and encodes a putative transcription factor. This may indicate that the generated SNP conferred the observed resistance and growth phenotypes via alterations in membrane composition or fluidity. To further investigate this we quantified ergosterol levels in the 3 strains. This revealed significant differences in ergosterol content between the strains. As shown in Fig. 5, despite variability, the parent strain (V130-15) contained significantly more ergosterol than the mutant (V130-15*unc*^167*^) and the strain in which the SNP was originally identified (V157-62) (p < 0.01 and p < 0.0001 respectively).

**Fig. 6:**
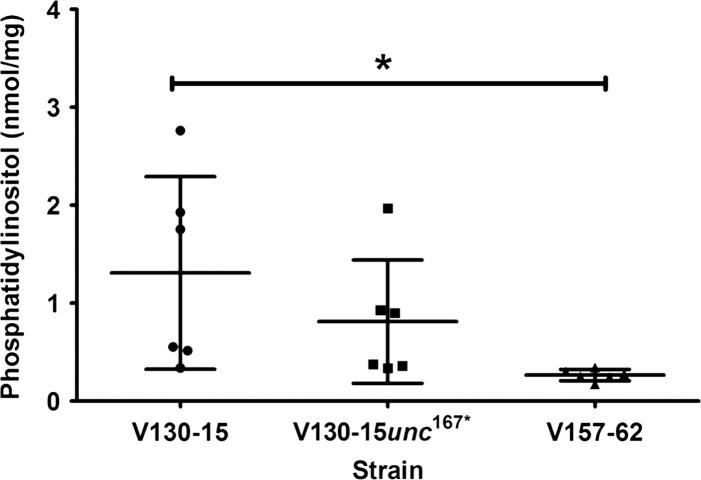
Quantification of phosphatidylinositol content of *A. fumigatus* strain V130-15, V130-15*unc*^167*^ and V157-62. Strains were cultured overnight in liquid glucose minimal media at 37 °C shaking at 200 rpm. Fungal mass was dried and ground in liquid nitrogen. Samples were subjected to chloroform-methanol extraction. Phosphatidylinositol (32:4) was then quantified using liquid chromatography-mass spectrometry (LC–MS). Data represents two independent extraction experiments, comprising 6 biological repeats per strain and 3 technical repeats per sample. Error bars represent ± SD (^*^p < 0.05; two-tailed Students T-test).

In order to assess whether this change was associated with alterations in lipid content, the phosphatidylinositol content of the strains was also assessed. As shown in Fig. 6, phosphatidylinositol content in V130-15 and the mutant (V130-15*unc*^167*^) were highly variable and no significant differences were identified. However, V157-62, possessed significantly less phosphatidylinositol in comparison to V130-15 (p < 0.05). This suggested that the SNP 167^*^ in AFUA_7G01960 affects ergosterol levels without significantly affecting phosphatidylinositol levels in *A. fumigatus*.

**Fig. 7:**
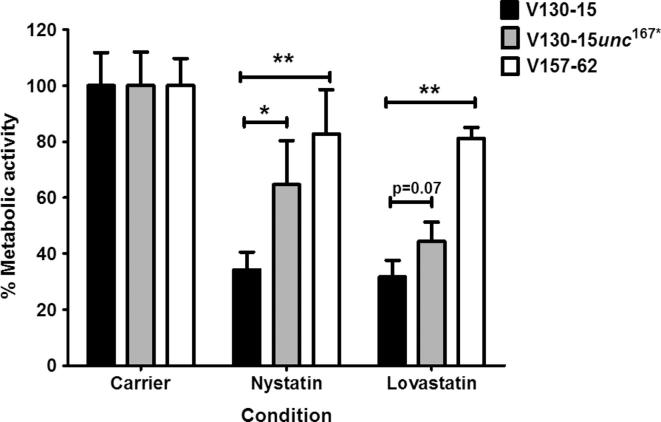
Susceptibility of *A. fumigatus* strains V130-15, V130-15*unc*^167*^ and V157-62 to lovastatin and nystatin. Metabolic activity of the strains was assessed using the XTT spectrophotometric assay after 48 h incubation with 16 µg/ml nystatin and 256 µg/ml lovastatin at 37 °C. Data represents two independent experiments with 3 repeats per experiment. Error bars represent ± SD (^*^p < 0.05; ^**^p < 0.01; two-tailed Students T-test).

In order to determine whether these differences in ergosterol levels were biologically relevant, resistance to nystatin (targeting ergosterol) and lovastatin (targeting HMG-CoA) was assessed. Resistance to statins was determined as a measure of metabolic activity after 48 h incubation with nystatin or lovastatin. As shown in Fig. 7, both the mutant (V130-15*unc*^167*^) and V157-62 showed increased resistance to 16 µg/ml nystatin and 256 µg/ml lovastatin. In comparison to V130-15, V157-62 showed significantly higher metabolic activity in the presence of nystatin and lovastatin (p < 0.01). In comparison to the parent V130-15, V130-15*unc*^167*^ showed a significantly higher metabolic activity in the presence of nystatin (p < 0.05), as well as in the presence of lovastatin although this difference was not statistically significant (p = 0.07). Therefore, the observed decreases in ergosterol content correlated with increased nystatin and lovastatin resistance, confirming their biological relevance.

## 4 Discussion

In this study, we used a CRISPR-Cas9 method to create single nucleotide modifications in clinical isolates of *A. fumigatus*. We used this approach to investigate the phenotypic impacts of specific SNPs that had been acquired during the course of recurrent and chronic infection. Firstly, we showed that a specific SNP in *svf1* affects the susceptibility of *A. fumigatus* to oxidative stress, inferring that *svf1* contributes to oxidative stress resistance. Secondly, we identified a SNP in an uncharacterised gene, AFUA_7G01960, which when recapitulated in a wild-type strain, resulted in increased azole resistance and decreased ergosterol content in the fungal membrane.

Investigating genetic changes using clinical strains is often challenging, as they possess wild-type *akuB* and therefore favour non-homologous recombination. Excitingly, CRISPR-Cas9 technology is revolutionising genome editing in fungi as it overcomes the challenges of non-homologous recombination (Ran et al., 2013). Recent publications have shown the successful use of CRISPR-Cas9 to perform genome editing in clinical *A. fumigatus* strains (Al Abdallah et al., 2017, Umeyama et al., 2018). In this study, we first reverted the G54R mutation to WT in *cyp51A*, to test whether the itraconazole resistance assigned to this mutation was lost without affecting the high-level posaconazole resistance. This expectation was confirmed, reinforcing the association of this mutation with itraconazole resistance but not high level posaconazole resistance (Chen et al., 2005, Diaz-Guerra et al., 2003, Howard et al., 2006, Mann et al., 2003, Nascimento et al., 2003, Xu et al., 2010). No other phenotypic changes were observed, suggesting minimal off target effects of CRISPR-Cas9 in *A. fumigatus* (Al Abdallah et al., 2018, Al Abdallah et al., 2017, Umeyama et al., 2018). The resistance mechanism conferring this high level posaconazole resistance in V157-39 remains unknown. However, it is possible that the non-synonymous SNPs previously identified in this isolate, influence this resistance (Ballard et al., 2018).

Recreation of the SNP 213^*^ in *svf1* resulted in increased sensitivity to oxidative stress. We hypothesise that this nonsense mutation causes truncation of the protein and probably (at least partial) loss of protein function, thereby leading to the observed oxidative stress sensitivity. In support of our hypothesis, the orthologue of *svf1* in *S. cerevisiae* has been shown to be required for survival in response to oxidative stress and mutants lacking this orthologue are unable to grow in the presence of oxidative stress (Brace et al., 2005). The 213^*^ SNP is particularly interesting as it was identified in an *A. fumigatus* isolate infecting a chronic granulomatous disease (CGD) patient. This patient group fails to generate a proper phagocytic respiratory burst due to defects in nicotinamide adenine dinucleotide phosphate (NADPH) oxidase, and hence their phagocytes fail to produce high levels of reactive oxygen species (Curnutte et al., 1974). Therefore, it is conceivable that the oxidative stress sensitivity conferred by this SNP did not impose a significant fitness or virulence defect in this specific host, as a proper oxidative stress response is not mounted.

In this study we also showed that recreation of the SNP 167^*^ in AFUA_7G01960 increased the resistance of this strain to itraconazole, resulted in a temperature dependent increase in growth rate, and a significant reduction in total ergosterol content. Interestingly, da Silva Ferreira et al., previously showed this gene to be upregulated upon voriconazole exposure (da Silva Ferreira et al., 2006). Based on our bioinformatics analysis, it can be hypothesised that AFUA_7G01960 encodes a putative transcription factor that promotes ergosterol synthesis and that the introduction of this SNP attenuates the activity of this putative transcription factor. The resulting reduction in ergosterol levels correlated with increased resistance to lovastatin and nystatin. Lovastatin inhibits HMG-CoA reductase, an enzyme early in the ergosterol biosynthetic pathway, and nystatin binds ergosterol (Harman and Masterson, 1957, Sirtori, 1990). Therefore, the reduced levels of ergosterol synthesis in the V130-15*unc*^167*^ and V157-62 strains probably explain their enhanced statin resistance. Ergosterol is known to regulate membrane stability and fluidity (Rodrigues, 2018). Therefore, the alterations in ergosterol content probably impacted membrane fluidity thereby mediating the temperature dependent effects on growth. These alterations also have potential to confer azole resistance, by altering membrane barrier function and as a result, intracellular accumulation of azoles (Lupetti et al., 2002).

In *Candida albicans* reductions in ergosterol levels have previously been associated with increased azole resistance (Kanafani and Perfect, 2008, Kelly et al., 1997, Kohli et al., 2002, Löffler et al., 2000, Mishra et al., 2008)*.* In addition, a collection of fluconazole resistant clinical *C. albicans* isolates were shown to possess defective sterol Δ^5,6^-desaturation, which resulted in alteration of membrane composition to include more 14α-methylfecosterol (Kanafani and Perfect, 2008, Kelly et al., 1997). The putative transcription factor identified in this study showed structural similarities to *upc2* in *S. cerevisiae*; a G888D SNP in *upc2* has previously been reported to result in increased levels of ergosterol and increased resistance (Ha et al., 2013, Yang et al., 2015)*.* Recently, decreases in relative ergosterol levels have been associated with increased azole resistance in *A. fumigatus* (Rybak et al., 2019)*.* In our study, using comprehensive lipidomics analysis, we were able to associate decreases in ergosterol levels to increased azole resistance in *A. fumigatus*. Intriguingly, decreases in ergosterol content did not result in higher MICs for amphotericin B. As amphotericin B is hypothesised to target ergosterol this was unexpected. However, the lack of an association between decreased ergosterol content and increased amphotericin B resistance, was recently also reported by others (Rybak et al., 2019). Most likely the reduced amount of ergosterol still results in sufficient binding of amphotericin B to the fungal cell membrane to exert its effect.

It has previously been reported that fungal cells grown in the presence of azoles contain decreased levels of ergosterol in comparison to control conditions (Arthington-Skaggs et al., 1999, Müller et al., 2018)*.* We hypothesise that strain V157-62 shows an adaptation to the presence of azole antifungals in-host and is now existing in an ‘azole adapted’ state. It is important to note that V157-62 contained lower levels of ergosterol in comparison to V130-15*unc*^167*^, lower levels of phosphatidylinositol in comparison to both V130-15 and V130-15*unc*^167*^ and a growth defect that was not observed in the created mutant. It is important to note the identified SNPs do not explain the full resistance profile of V157-62. V157-62 has an M220R SNP in *cyp51A*, which does not explain the observed posaconazole or voriconazole resistance (Chen et al., 2005, Garcia-Effron et al., 2005). In addition, the identified 167^*^ SNP in AFUA_7G01960, was shown to not confer posaconazole or voriconazole resistance. It can be hypothesized that a complex interplay of various SNPs confers the overall resistance phenotype; it is of importance to unravel the contributions of individual SNPs in order to understand the overall mechanisms. V157-62 possesses ∼29 additional non-synonymous SNPs in comparison to V130-15 (Ballard et al., 2018). It is highly likely that these SNPs are also contributing to the overall resistance profile as well as the differences in phosphatidylinositol and ergosterol levels.

## 5 Conclusions

In summary, clinical isolates are exposed to numerous known and unknown stress factors, underscoring the diversity and complexity of the adaptation process involving various pathways. Genome editing systems such as CRISPR-Cas9 will help to gain detailed insights into the complex interplay of SNPs involved in in-host adaptation in *A. fumigatus.* Using CRISPR-Cas9 we have unravelled the function of specific in-host acquired SNPs in clinical *A. fumigatus* isolates. We have identified a specific SNP (167^*^ in AFUA_7G01960) that was acquired in-host during infection and that compromises azole therapy. This SNP was associated with non-*cyp51A* mediated antifungal resistance. Further insight into the underlying mechanisms conferring resistance by this SNP were obtained by lipidomics analysis, which showed strains possessing the SNP to have decreased ergosterol levels. As this SNP was revealed in a clinical isolate, which acquired azole resistance in-host, we have identified a clinically relevant novel mechanism of azole resistance in *A. fumigatus*.

## Figures and Tables

**Table A.1:** Sequences of primers used for plasmid construction, donor DNA and SNP verification.

Target	Primer use	Primer ID	Fw/Rv	Sequence (5′-3′)
AFUA_4G06890	Plasmid construction	oEB001	Fw	CTTCACCATCAGTTACAGGATTGATCCCGGAGCTTCTCAGACCAAGC
		oEB004	Rv	TAACTGAGACGAGCTTACTCGTTTCGTCCTCACGGACTCATCAGTCAGTTCGGTGATGTCTGCTCAAGC
		oEB003	Fw	TGAGGACGAAACGAGTAAGCTCGTCTCAGTTACAGGATTGATCCCGTTTTAGAGCTAGAAATAGCAAGT
		oEB002	Rv	TCCGGGATCAATCCTGTAACTGATGGTGAAGAGCTTGACATTGGGGA
	Donor DNA/SNP validation primers	oEB020	Fw	CTCTAATCCTCGGGCTCACC
		oEB021	Rv	CTCCATCAGCTTGGAATTGG

AFUA_5G11820	Plasmid construction	oEB075	Fw	CTTCACCTGTACAACTTCATGTTCGATGGGAGCTTCTCAGACCAAGC
		oEB078	Rv	TTGTACGACGAGCTTACTCGTTTCGTCCTCACGGACTCATCAGGTACAACGGTGATGTCTGCTCAAGC
		oEB077	Fw	AGGACGAAACGAGTAAGCTCGTCGTACAACTTCATGTTCGATGGTTTTAGAGCTAGAAATAGCAAGT
		oEB076	Rv	TCCCATCGAACATGAAGTTGTACAGGTGAAGAGCTTGACATTGGGGA
	Donor DNA/SNP validation primers	oEB081	Fw	AGGGAACTGCGTACACCATC
		oEB082	Rv	GAGTATGACGGGGTCTGGAA

AFUA_7G01960	Plasmid construction	oEB070	Fw	CTTCACCCTCTAATGCGAAAGCTATGTTGGAGCTTCTCAGACCAAGC
		oEB073	Rv	TTAGAGACGAGCTTACTCGTTTCGTCCTCACGGACTCATCAGTCTAATCGGTGATGTCTGCTCAAGC
		oEB072	Fw	AGGACGAAACGAGTAAGCTCGTCTCTAATGCGAAAGCTATGTTGTTTTAGAGCTAGAAATAGCAAGT
		oEB071	Rv	TCCAACATAGCTTTCGCATTAGAGGGTGAAGAGCTTGACATTGGGGA
	Donor DNA/SNP validation primers	oEB062	Fw	GCTCATTTTTGTGGGCAAAT
		oEB063	Rv	ACGTATCTCGGTTGCAGCTT

## Appendix A

See Fig. A.1 and Table A.1.
